# Supplementary material for: Directional information flow as a tool for analyzing protein allostery
Source: bioRxiv. 2026 Jun 23:2026.06.22.733418. Preprint. [Version 1] doi: 10.64898/2026.06.22.733418 (PMC13320769; doi:10.64898/2026.06.22.733418)
Supplement: Supplement 1 [file media-1.pdf]

Supplemental Information Title: Directional information flow as a tool for analyzing protein allostery

Authors: Remy A. Yovanno and Albert Y. Lau

Supplemental information includes:

Figures S1-S4

Additional data tables can be found in TE_supplemental_data_tables.xlsx

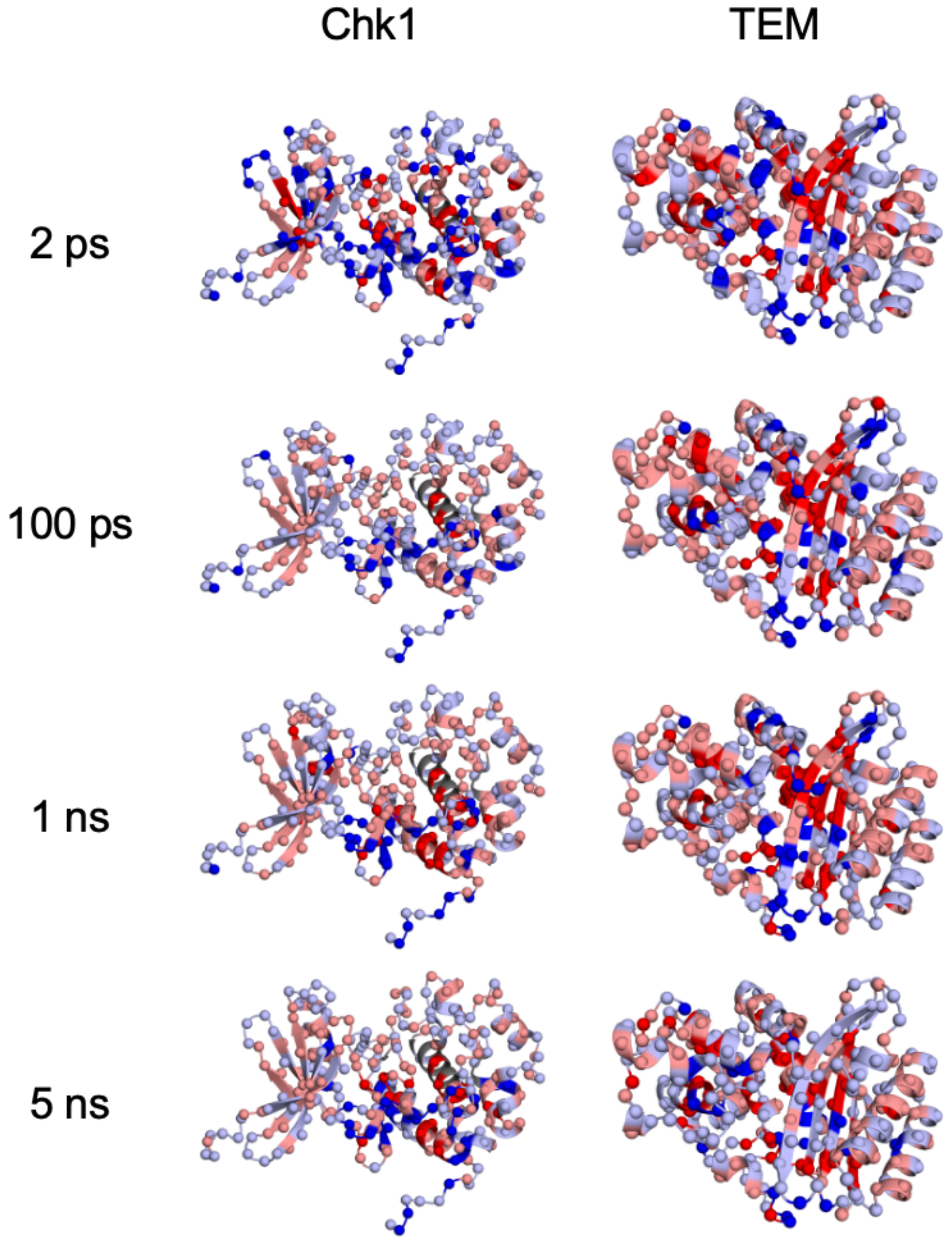

Fig. S1: Effect of the selection of time lag τ* on transfer entropy flux. Transfer entropy flux (TE flux) was visualized for a range of different time lags: 2 ps, 100 ps, 1 ns, and 5 ns.

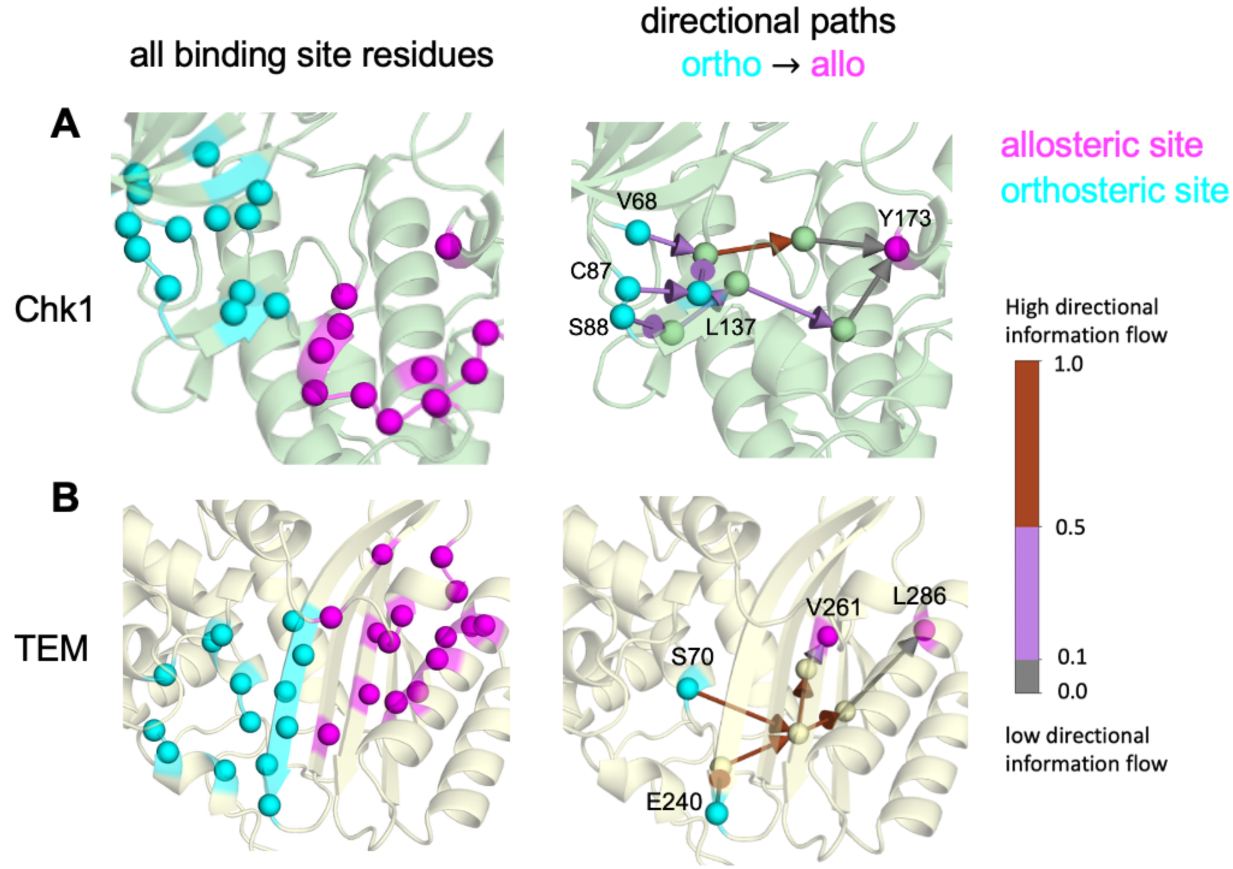

Fig. S2: Directional paths from orthosteric site residues to allosteric site residues in **(A)** Chk1 and **(B)** TEM. A complete list of Δ values ranking directional paths can be found in **TE_supplemental_data_tables.xlsx**.

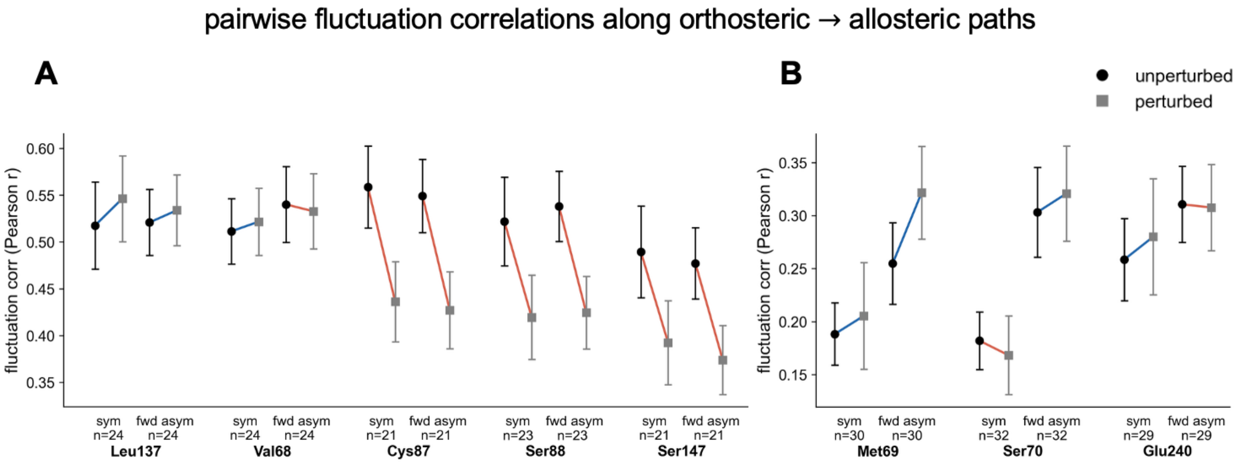

Fig. S3: (A-B) Analysis of pairwise correlations along paths from perturbed orthosteric residues to the allosteric site. Pairwise fluctuation correlations were computed for residue pairs in the path ensemble with the highest forward TE asymmetry ($TE_{forward}$ – $TE_{reverse}$, top 25%) and near-zero TE asymmetry ($|TE_{forward} - TE_{reverse}| \approx 0$, bottom 25%) for each orthosteric site perturbation for **(A)** Chk1 and **(B)** TEM (perturbed residue name shown in bold). Error bars represent the SEM across edges within each group. Differences between unperturbed (black circles) and perturbed (gray squares) dynamics were visualized for forward asymmetric and near-symmetric pairs. Red and blue connecting lines indicate decreased or increased correlations upon perturbation, respectively.

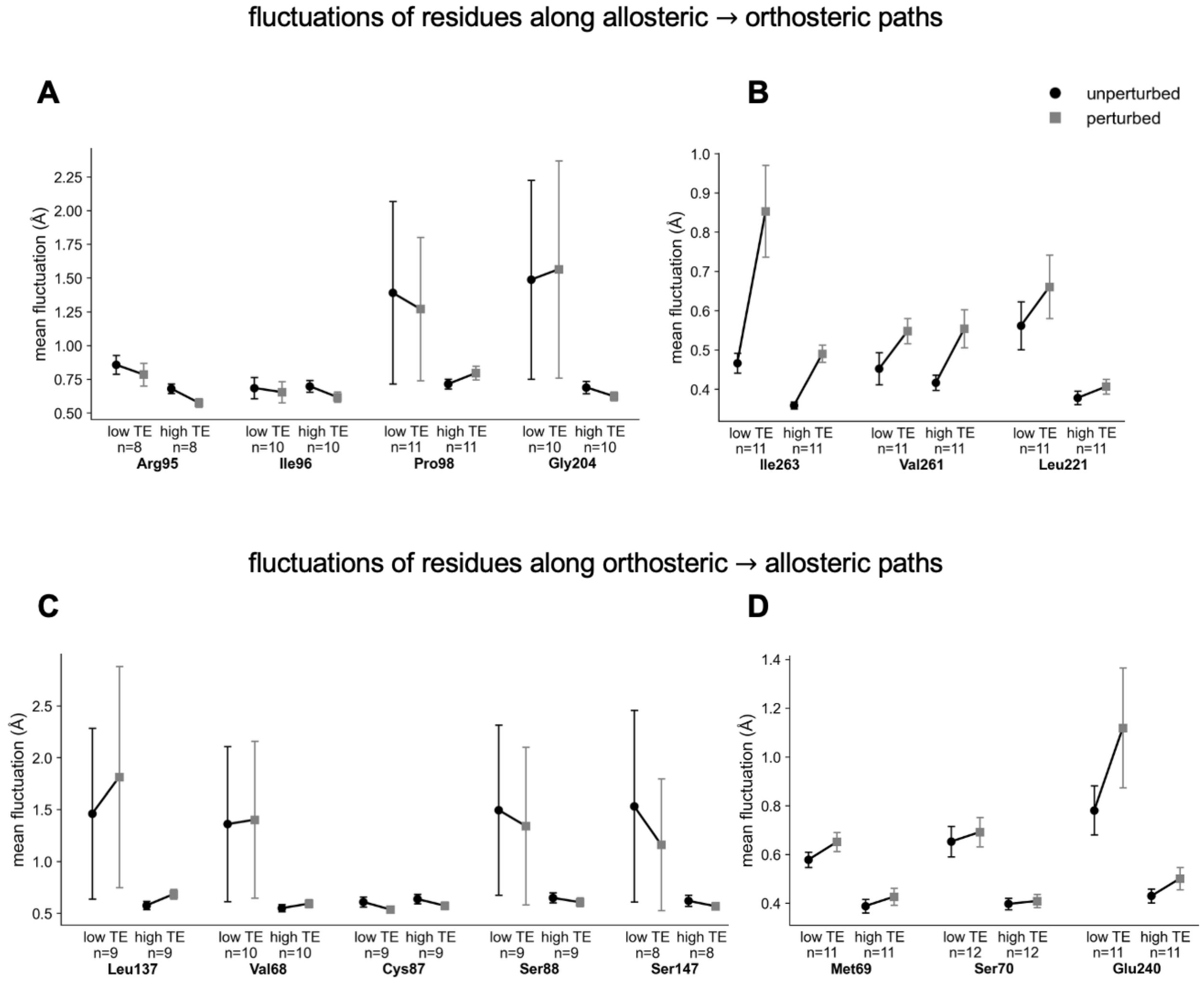

Fig. S4: Analysis of per-residue fluctuations along paths between perturbed residues and the opposite binding site. Per-residue fluctuations were computed for path residues with both high and low incoming TE. **(A-D)** Mean fluctuations are plotted for both high TE (top 25% of path residues) and low TE (bottom 25% of path residues) from unperturbed (black circles) and perturbed (gray squares) trajectories. Error bars represent the SEM across residues in the low and high TE groups. (**A-B)** Fluctuations are shown for residues on paths from allosteric perturbations to orthosteric site residues for **(A)** Chk1 and **(B)** TEM. **(C-D)** Fluctuation analysis is shown for residues on paths from orthosteric perturbations to allosteric site residues for **(C)** Chk1 and **(D)** TEM.
